# A Miniaturized, Programmable Deep-Brain Stimulator for Group-Housing and Water Maze Use

**DOI:** 10.3389/fnins.2018.00231

**Published:** 2018-04-12

**Authors:** Richard C. Pinnell, Anne Pereira de Vasconcelos, Jean C. Cassel, Ulrich G. Hofmann

**Affiliations:** ^1^Laboratoire de Neurosciences Cognitives et Adaptatives, Faculté de Psychologie de Strasbourg, Université de Strasbourg, Strasbourg, France; ^2^Section of Neuroelectronic Systems, Neurosurgery, Medical Centre, University of Freiburg, Freiburg im Breisgau, Germany; ^3^Faculty of Medicine, University of Freiburg, Freiburg im Breisgau, Germany; ^4^Freiburg Institute for Advanced Studies, University of Freiburg, Freiburg im Breisgau, Germany; ^5^University of Strasbourg Institute for Advanced Study, University of Strasbourg, Strasbourg, France; ^6^LNCA, UMR 7364, Centre National de la Recherche Scientifique, Strasbourg, France

**Keywords:** deep brain stimulation, portable device, stimulator, waterproof, implant, group-housing, rat behavior, water maze

## Abstract

Pre-clinical deep-brain stimulation (DBS) research has observed a growing interest in the use of portable stimulation devices that can be carried by animals. Not only can such devices overcome many issues inherent with a cable tether, such as twisting or snagging, they can also be utilized in a greater variety of arenas, including enclosed or large mazes. However, these devices are not inherently designed for water-maze environments, and their use has been restricted to individually-housed rats in order to avoid damage from various social activities such as grooming, playing, or fighting. By taking advantage of 3D-printing techniques, this study demonstrates an ultra-small portable stimulator with an environmentally-protective device housing, that is suitable for both social-housing and water-maze environments. The miniature device offers 2 channels of charge-balanced biphasic pulses with a high compliance voltage (12 V), a magnetic switch, and a diverse range of programmable stimulus parameters and pulse modes. The device's capabilities have been verified in both chronic pair-housing and water-maze experiments that asses the effects of nucleus reuniens DBS. Theta-burst stimulation delivered during a reference-memory water-maze task (but not before) had induced performance deficits during both the acquisition and probe trials of a reference memory task. The results highlight a successful application of 3D-printing for expanding on the range of measurement modalities capable in DBS research.

## Introduction

In animal behavioral studies, deep-brain stimulation (DBS) research has traditionally relied on the use of a cable tether, for connecting an awake animal to the stimulating hardware. Not only does this require a purpose-built arena for each animal for accommodating such a tether, but such a method can reduce animal mobility and increase stress (Tang et al., 2004). Also, the risk of cable breakages, snagging or entanglement is present, and further exacerbated over long periods of time. To circumvent these issues, numerous portable stimulators have been developed for animal use, including head-mount systems (Arfin et al., 2009; Forni et al., 2012; Hentall, 2013; Kouzani et al., 2013, 2016), back-mount systems use using a Velcro jacket (Song et al., 2006; Feng et al., 2007; Ewing et al., 2013), and implantable systems (Millard and Shepherd, 2007; de Haas et al., 2012). While these devices are successful in granting the operator increased flexibility with regards to the experimental design and arena selection, there are behavioral paradigms with which these devices cannot be utilized.

Until now, such experiments involving portable stimulators have been limited mainly to individually-housed rat use, due to the risk of damage to the device, implant, or wound, through various social behaviors such as grooming, playing and fighting. The benefits of group-housing rats include a normalization in many behavioral and physiological effects that would otherwise occur in healthy rats, including weight gain (Levitsky, 1970; Fiala et al., 1977; Pérez et al., 1997; Lopak and Eikelboom, 2000; Pinnell et al., 2016), stress-induced FOS activity (Westenbroek et al., 2003), as well as heart-rate and blood pressure changes (Sharp et al., 2002). This becomes important when chronic stimulation paradigms are utilized, which may involve weeks of social isolation (e.g., Forni et al., 2012). Being able to co-house animals during prolonged periods of stimulation, may offer a way to normalize stress-induced behavioral and physiological deficits that may otherwise interfere with the parameters under study.

Another test condition that proves problematic for portable stimulators is water-maze use. In most studies that have assessed the effects of DBS using a water-maze, stimulation has been carried out either before (Hamani et al., 2011; Zhang et al., 2015; Hescham et al., 2017) or after (Ruiz-Medina et al., 2008; Schumacher et al., 2011; Jeong et al., 2014) the maze task. Several attempts at providing waterproof EEG recording or stimulation have been made using a cable tether (Hollup et al., 2001a,b; Fyhn et al., 2002; McNaughton et al., 2006; Korshunov and Averkin, 2007; Sweet et al., 2014). While mobility deficits cannot be ruled out with such a method, the use of an overhead cable has the potential to cause artifacts on overhead video-tracking systems. Alternatively, animal implantable stimulators can offer the inherent ability of being waterproof, but they cannot undergo battery changes, their parameters are fixed, and they may cause discomfort to the animal during tasks that require locomotion or swimming.

To address these issues, a portable DBS device was developed for head-mount use in rats, by combining a 3D-printed device housing with a miniature PCB assembly. The device, its battery and housing weighs 2.7 g, and offers protection from both the environment and from other rats. Furthermore, the device can be utilized inside a water maze, using a magnetic switch to activate/deactivate the device as needed.

Generally speaking, portable stimulators do not match the performance or functionality of their tethered counterparts, owing to the limited size of the system and its battery, and the necessity to employ space-saving and low-power techniques. While there exist numerous portable stimulators that can function for well over 10 days (e.g., Millard and Shepherd, 2007; Harnack et al., 2008; Forni et al., 2012; Hentall, 2013), the majority of portable stimulators may lack either a high compliance voltage (>10 V), voltage regulation, charge-balancing, biphasic pulses, adjustable parameters, or a combination thereof (Millard and Shepherd, 2007; Harnack et al., 2008; Arfin et al., 2009; de Haas et al., 2012; Hentall, 2013; Kouzani et al., 2013, 2016). A high compliance voltage (>10 V) can ensure a stable constant current that is maintained through a range of tissue types and electrode impedances, and regulation ensures that it is fixed throughout the duration of the system's battery life. Such voltages are more likely to be present on larger devices weighing over 5 g (Harnack et al., 2008; Zhou et al., 2012; Ewing et al., 2013), due to the additional space for accommodating voltage amplification stages and a larger battery. Charge-balanced biphasic pulses can offer both the ability to normalize net charge inside the brain following a stimulus pulse, as well as causing less tissue damage when compared to monophasic pulses (Merrill et al., 2005). Furthermore, the ability to fully configure the device's parameters (pulse-width, frequency, current intensity, and pulse mode) can enhance the system's versatility between experiments, while paving the way toward repeated-measures experimental designs involving multiple stimulus paradigms. The current system aims to provide some of the expanded functionality of tethered systems, by offering 2 charge-balanced biphasic channels with a high compliance voltage (>12 V) and a flat pulse profile, a magnetic on/off switch, a programmable LED, and a wide range of programmable stimulus parameters and pulse modes.

The device was verified in two rat behavioral experiments that investigated the effects of nucleus reuniens DBS. Such a structure was investigated due to its dense, bi-directional connections with the prefrontal cortex and hippocampus, and its possible role in learning and memory (see Cassel et al., 2013, for a review Cassel et al., 2013). In the first experiment, animals (*n* = 44) were trained in a place-learning variant of the Morris Water Maze (MWM) task, during which they had received either theta-burst-stimulation (TBS; before or during acquisition) or sham-stimulation. TBS involves a high-frequency burst of pulses (e.g., 500 Hz) delivered at a theta frequency (e.g., 7 Hz), to provide a means of mimicking the naturally-occurring theta rhythm. These parameters were chosen to facilitate or disrupt the naturally occurring theta signal in the nucleus reuniens, an area suspected for providing modulation of prefronto-hippocampal interactions (Griffin, 2015). Animals stimulated inside the maze (but not before) were seen to display mild performance deficits both during the acquisition and probe sessions, as observed by measures of the time spent in the target quadrant, mean distance to target, and the swim efficiency. The second experiment verified the ability of the devices to be used with pair-housed rats (*n* = 14), in a chronic high-frequency stimulation paradigm. No malfunctions, leakages, or problems were reported in any of the devices throughout the entire test period; highlighting its capability as a robust and versatile device for expanding the range of behavioral paradigms for pre-clinical DBS research.

## Materials and methods

### Device design

**Figure 1:**
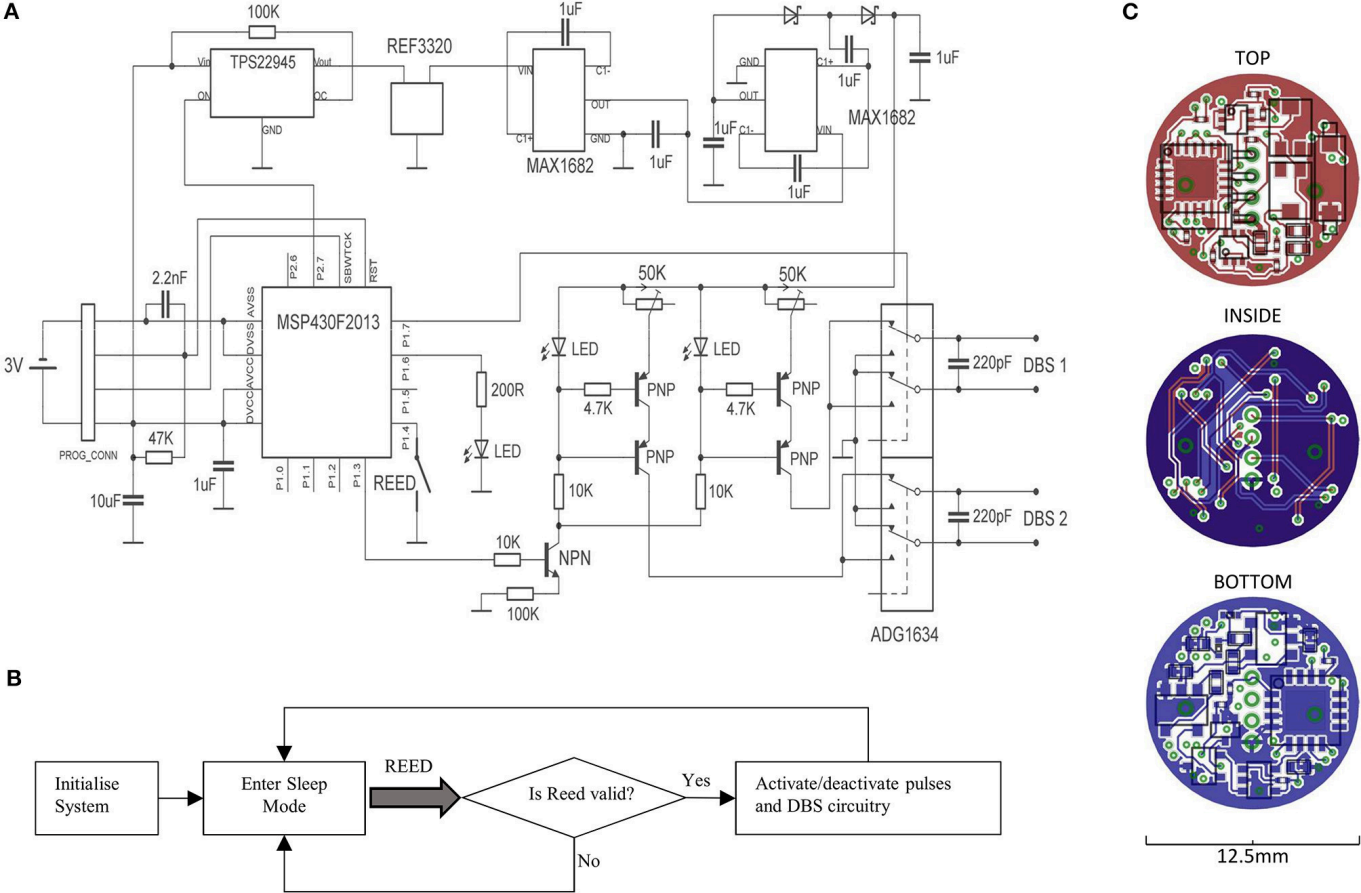
A circuit diagram for the portable stimulator is shown **(A)**, alongside a flowchart for the firmware **(B)**. The layout of copper tracks, pads, vias, and components are shown for the top, bottom, and internal layers of the PCB **(C)**.

A circuit diagram is shown, alongside its corresponding PCB layout that depicts the top, bottom and internal copper layers (Figures 1A,C). During operation, a microcontroller (MSP430F2013; Texas Instruments) is used to generate pre-programmed voltage pulses, which in turn gates the flow of constant current through a transistor switch. The current is generated by arranging a PNP transistor pair alongside an LED, such that the LED maintains a fixed voltage reference across a variable resistor. The system supports a current range of 20 μA−2 mA, if the compliance voltage limit (12.29 V) is not exceeded. As such, two LED's are active during stimulation, which provides visual feedback to the operator. The constant current pulses are interfaced to a quad single-pole double-throw (SPDT) digital switch (ADG1634; Analog Devices), which can switch the direction of the current across the channel pair, thereby producing biphasic pulses. The switch is also configured to connect stimulus electrodes to ground immediately following a stimulus pulse, which can enable charge balancing for monophasic pulses, while improving it somewhat for biphasic pulses. The compliance voltage for the constant-current circuitry is generated amplifying a fixed/rectified 2.048 V voltage by 6, using 2 voltage doublers connected in series (MAX1682; Maxim Integrated Products); the second of which is configured to triple the voltage by inclusion of a Schottky-capacitor rectifier. The voltage reference (REF3320; Texas Instruments) ensures that the compliance voltage is fixed at 12.29 V throughout the duration of the battery life.

A miniature Reed switch is provided (RI-80 SMD; Comus, USA), for allowing the system to be magnetically activated/deactivated. During activation, a timer-controlled interrupt is used for generating timed stimulus pulses (Figure 1B). During deactivation, the microcontroller enters its lowest power state (LPM4), and the voltage reference, DC amplification, and constant-current generators are deactivated via a digital power switch (TPS22945; Texas Instruments, USA). The system was programmed to accept only magnets held in place from 0.8 to 1.2 s, to prevent unwanted activation/deactivation by accidental means.

All the device components and integrated circuits were distributed onto 2 sides of a 1 mm × 12.5 mm diameter, circular 4-layer PCB (PCB-Pool; Beta Layout GmbH, Germany). The smallest available packages were chosen for every component (while meeting electronic requirements e.g., capacitor voltage ratings), including 0201 passives and quad-flat-pack (QFN) integrated circuits; and the design was repeatedly optimized to provide a maximum reduction in PCB space. Circuit track widths are 125 μm, and the vias are 0.2 mm. Miniature custom connectors were used for both the programming connectors, and the DBS terminals (Fischer's Elektronic, Germany), the latter of which are connected to the PCB by a 2 cm pair of twisted insulated wire strands. All the device components were covered with a layer of UV-curing adhesive (Loctite, USA).

Prior to use, the system is programmed using an MSP-FET programmer/debugger tool (Texas Instruments, USA), with the chosen stimulus parameters. The current intensity is set by placing a 20 KΩ fixed-value resistor across the DBS output terminals, and inferring the current from the voltage drop across the resistor, using a digital oscilloscope.

### 3D-printed head-cap

A 3D-printed head cap (12.4 × Ø19.2 mm; 1.1 g) was designed in Solid Edge ST6 (Siemens PLM Software) and printed with clear ABS plastic using a 3D printer (Ultimaker 2; Ultimaker). The cap was designed to enclose the portable stimulator and its battery, and to attach to a 3D-printed skull socket (Pinnell et al., 2016) using two electronic self-tapping screws (M1.4 × 4 mm; Phillips). The interior of the cap was shaped as appropriate, to fit to the contours of the device and its battery.

### Electrodes

LFP electrodes consisted of a single strand of 150 μm diameter (125 μm bare) polyimide-coated stainless-steel wire (005SW/2.0 S; Plastic's One, USA), whereas the bipolar stimulating electrodes consisted of two strands twisted together using a dental drill. For both electrode types, 200 μm of Polyimide was scraped from the electrode tip using a scalpel, for providing a suitable contact area for stimulation or recording. After fabrication, both electrode types were immersed into saline, and the stimulator was used to send a 90% duty cycle, 1 mA current through them. The resulting hydrogen bubbles that formed (via hydrolysis) could expose any breaches in the material resulting from assembly, as well as any connectivity problems such as short-circuits. Immediately prior to surgery, bipolar stimulating electrodes (measured at < 10 KΩ impedance) were further tested by sending 200 μA pulses through them, and observing the voltage drop across them. Any electrode that fell outside a 2–4 V median range were discarded.

### Surgery

All experiments were conducted in adherence to the regulations and guidelines, as specified by the international (NIH publication no 86–23, revised 1985) laws and policies, and the European Committee Council Directive of November 24th, 1986 (86/609/EEC). All protocols were approved by the Animal Care Committee of the University of Freiburg (permit 35-9185.81/G-13/97), and the French Department of Agriculture, where appropriate.

Male Long Evans rats (280–300 g; *n* = 44) were anesthetized with ketamine/xylene (0.23 ml.kg^−1^ i.p.; 23% Xylazine; 38% Ketamine; 38% Saline), and were then secured into a stereotaxic frame (David Kopf Instruments, USA). Rats were implanted with a single bipolar stimulating electrode into the midline thalamus (AP-2.3; ML-1.6; DV-7.4 mm, at 13° inclination), as measured relative to the skull surface at Bregma. The electrode connectors were encapsulated inside a 3D-printed implant, which was attached to the skull using two stainless steel mounting screws (0–80 × 1/8; Plastics One; USA). The rear mounting screw functioned also as a reference electrode, for EEG recordings. An additional 3 mounting screws were applied around the skull perimeter to provide additional support. The enclosure was then filled with dental cement (Palapress; Heraeus Holding GmbH; Germany). In the chronic group, female Sprague Dawley rats (280–300 g; *n* = 14) underwent the same procedure, but with an additional LFP electrode implanted into the medial prefrontal cortex (AP+3.0; ML-0.7; DV-3.5 mm), and dCA1 region of the hippocampus (AP-3.6; ML-2.5; DV-2.6 mm).

Animal breathing and reflexes were checked throughout the surgery period, and animals were examined daily for signs of distress or discomfort. Sprague Dawley rats received an analgesic during immediately before surgery, and for the next 4 days afterwards (Carprieve, 1 ml kg^−1^s.c.; Norbrook, UK). Long-Evans rats were alternatively provided with a general anesthesia (Duphamox, 300 μl i.m.; Zoetis, USA) and local anesthesia (Lidocaine, 200 μl s.c.; Ceva Santé, France) before the surgery.

### Water-maze DBS

A MWM (1.6 m diameter) was situated in a diffusely-lit room with high-contrast extra-maze cues surrounding the walls. The water was rendered opaque using skimmed milk powder, and a thermometer was used to ensure a water temperature of 21°C. Rats were trained on a reference memory paradigm, consisting of an initial day of habituation, followed by 8 days of acquisition. During habituation, rats underwent 4 trials in which to locate a visible platform (11 cm, painted black, 1 cm above the water surface in the SE quadrant), whereby the starting position was randomized around the edge of the pool. A curtain was provided around the pool during this session, to obscure external cues. During acquisition, all rats underwent 4 trials/day in order to locate a hidden platform using external cues. A transparent platform was placed in the NW quadrant of the maze, and was submerged 2 cm below the surface of the water. The starting location was varied daily between the N, S, E, and W locations. For both session types, rats were given 1 min to swim to the platform location, after which they were left there for 10 s. Rats that did not reach the platform within 60 s were guided there by the experimenter and left there for 10 s. Rats were always placed into the maze facing the wall, and their test order was randomized for each day. Probe trials were given on days 3 and 6, which took place immediately prior to the day's acquisition training. In this session, the platform was removed, and animals were released from the SE quadrant, and left to swim for 60 s.

Long-Evans male rats were divided into the following groups: Sham stimulation (SHAM; *n* = 21), TBS before (BEF; *n* = 11), or TBS during (DUR; *n* = 12). Rats in all groups were affixed with a portable stimulator, 30 min prior to starting the task. Prior to attachment, a small amount of petroleum jelly (Vaseline) was applied to the inside of the device housing, to provide additional waterproofing. Stimulation was activated during the 30-min period prior to the task (BEF group), or during the MWM task (DUR group). Stimulation was not provided in the DUR group during any of the probe sessions. Animals in the SHAM group did not undergo stimulation at any point in the test, but they carried the devices in all test sessions. The stimulus pulses were delivered in 7 Hz bursts, each consisting of 16 × 200 μA biphasic pulses delivered at 500 Hz, and 100 μS pulse-width. The 7 Hz burst frequency was chosen to match a pre-recorded theta-frequency inside the nucleus reuniens during mobility (exactly 7 Hz).

Following each session, rats were gently dried with a towel, and were returned to their home cages whereby the portable devices were removed. Each recovered device was checked using an oscilloscope to verify that it could still deliver stimulus pulses at the correct settings. Numerous parameters were recorded during the test sessions using a video tracking system (Smart; Panlab), including the rat's position, latency, path length, quadrant time, average distance to target, and Whishaw's Index (a percentage measure of swim path traveled between a straight line connecting the start and goal locations, representing swim efficiency).

### Chronic DBS

Rats were pair-housed for 8 days, during which they had received DBS on days 3–7. Stimulation was activated in the STIM rats (*n* = 7; 130 Hz, 90 μS/phase pulse-width, 50 μS inter-pulse spacing, 200 μA biphasic) for 1 h, at 12 p.m. each day. Rats underwent recordings of EEG and mobility before and after this period, using a wireless recording system (W32; Multichannel Systems) and a video-tracking system (Cinelab; Plexon). During stimulation sessions, the status of the animals was monitored in another room using a camera mounted above the cages (Hero 3; GoPro).

### Statistics and representation

All data was imported into Matlab (Mathworks), for representation and statistical comparisons. Statistics in the MWM task utilized 2-way ANOVA's, looking at effects of session number (1–8) and group type (SHAM; BEF; DUR). Probe-trial differences used a 1-way ANOVA (looking at all groups). Student's *T*-tests were utilized for *post-hoc* comparisons between sham and stimulus groups.

For MWM swim position representation, the paths of each group were combined for a particular session, and converted to a normalized, 2D histogram. Each tracking point was converted to a 10 cm diameter circle prior to this, for better highlighting the group position preference.

## Results

### Device capabilities

**Figure 2:**
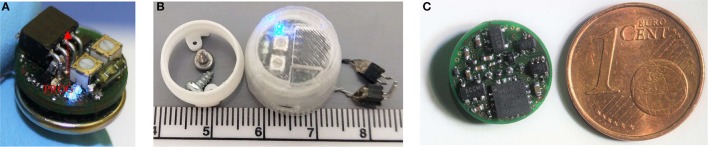
Photographs of the portable stimulator. The top side is shown, complete with a battery, its programming port (highlighted), potentiometers, reed switch and DBS LED's **(A)**. The system was utilized with a 3D-printed waterproof cap, which mounts onto a skull-implanted electrode socket **(B)**. The underside of the system is shown, alongside a coin for scale; highlighting the high component density **(C)**.

**Figure 3:**
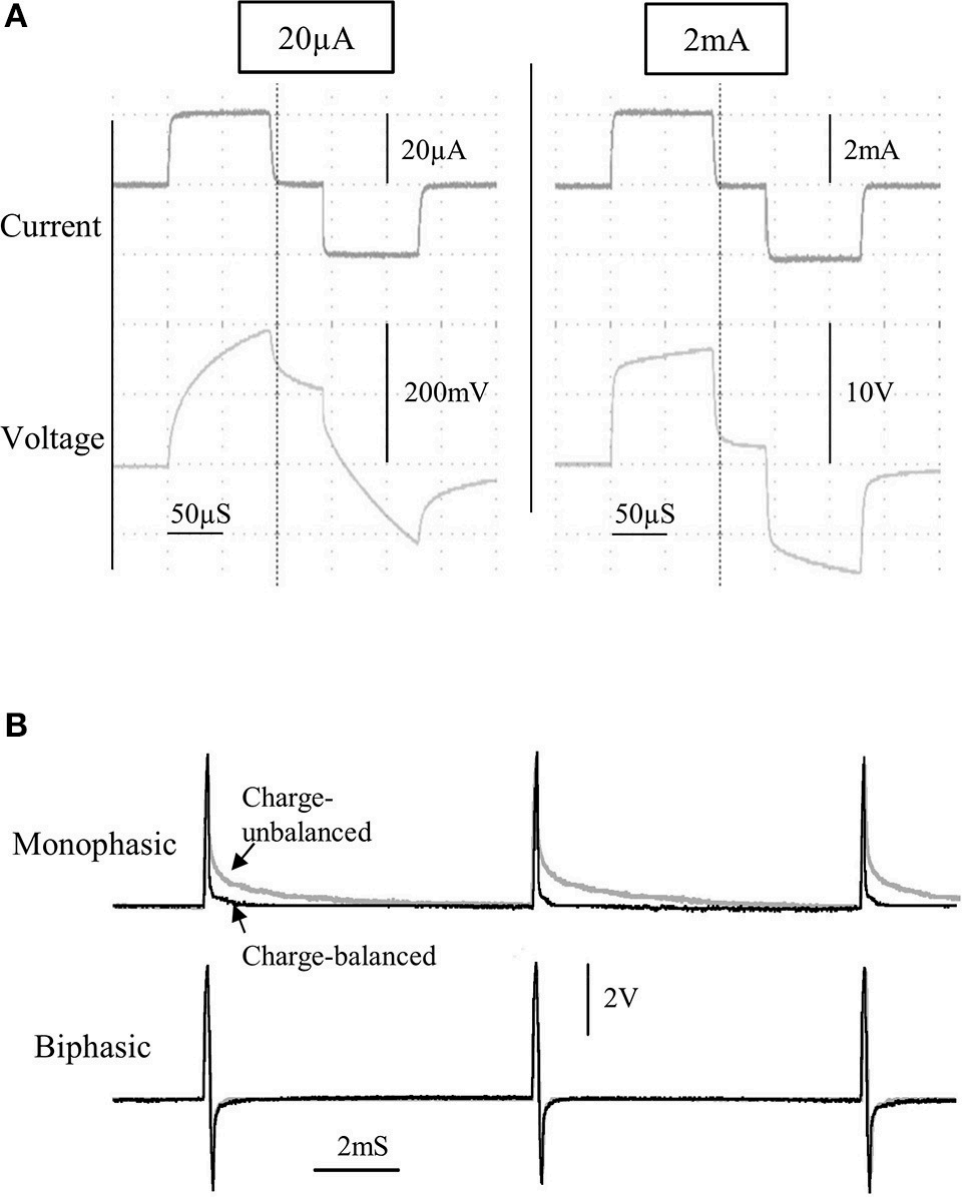
The current and voltage of the stimulus pulses was observed for a wide range of currents, from 20 to 2,000 μA, for a bipolar stimulating electrode immersed in saline **(A)**. A comparison between charge-balanced (black) and charge un-balanced (gray) monophasic/biphasic voltage waveforms is shown using 200 μA pulses **(B)**. Note that passive charge balancing is more noticeable with monophasic pulses, due to the increased charge build-up that would otherwise occur.

The portable stimulator (Figure 2) features two bipolar, charge-balanced channel pairs for DBS, with a 12 V compliance (see Table 1 for a full list of parameters). Numerous parameters can be programmed for use through a 4-pin micro connector, including the pulse mode (monophasic or biphasic), frequency (0.1-5,000 Hz) or the pulse-width (10 μS−100% duty cycle). Pulse trains can be selected as a fixed frequency (e.g., 130 Hz) or can employ a dual-frequency bursting pattern (e.g., 7/500 Hz theta-burst stimulation). The constant current is adjusted by manually turning potentiometers on the device (12 V compliance, delivering 20 μA−2 mA as tested in saline; see Figure 3).

**Table 1:** A comparison between the attributes of the proposed, and existing recent stimulators.

**Parameter**	**Proposed**	**de Haas et al., 2012**	**Forni et al., 2012**	**Kouzani et al., 2013**	**Hentall, 2013**
Dimensions	12.5 mm diameter × 5 mm	8 mm diameter × 30 mm	15 × 28 × 7 mm	12 mm diameter × ?	18 × 8 × 7 mm
Weight/w. battery	0.8/2.8 g (battery + housing)	?/2.1 g	6.5/7.4 g	5.1 g	?/2 g
No. channels	2 monophasic or 2 biphasic	2 biphasic	1 monophasic	1 monophasic	1 monophasic
Pulse shape	Flat	Non-flat	Flat	Non-flat	Non-flat
Compliance voltage	12 V (fixed)	4.65 V (battery)	4.5–6 V (battery)	1.8–3 V (battery)	34 V (fixed)
Pulse-width	10 μS−100% duty cycle	60 μS	0–80 μS	90 μS	100–1,000 μS/phase
Frequency	0.1–5,000 Hz	131 Hz	0–130 Hz	130 Hz	8, 16, 24 Hz
Inter-pulse interval	0–pulse period	200 μS	n/a	n/a	n/a
Battery life (DBS ON)	30 h	10 h	7 days	10 days	42 days
Other	-Programmable -Magnetic switch -Status LEDs -Waterproof -Social-proof	-Magnetic switch -IR LED			-Programmable -Magnetic switch -IR status LED

*A question mark is placed where information is unavailable. Note that the compliance voltage is shown instead of constant-current intensity, since the latter depends on the compliance voltage, and the brain and electrode impedances*.

The device can be powered down to an ultra-low power stage during inactivation, and subsequently reactivated by placing a magnet near the device. During a low-power stage, the device consumes approximately 35 μA, and can theoretically remain in this stage for many months. The magnetic activation/deactivation parameters are programmed, such that the device activates/deactivates when a magnet is held in place for 1 ± 0.2 s. This “time window” of activation reduces the likelihood of the device being accidentally activated/deactivated by a magnetic object. In addition to two LED's that are active during DBS, a separate status LED provides the user with a feedback regarding the magnetic activation/deactivation, and can be programmed with a variable brightness and a flash sequence during normal use. Finally, pulses can be programmed to be continuous, finite-duration (e.g., 30 s), and/or to begin after a fixed time duration following magnetic activation.

The device is housed inside a 3D-printed protective cap (Figure 2) during use, and can be removed and reattached to a surgically-affixed skull-socket. This provides for a strong and stable device attachment, which can withstand various rat social behaviors including grooming, playing and fighting. The device utilizes a single removable CR1225 battery (0.9 g), which is situated directly underneath the device inside the protective cap. This battery provides approximately 30 h of constant DBS, when tested in saline at the following parameters: 2 channels, 130 Hz, biphasic, 90 μS/phase, 200 μA current, 50 μS inter-pulse interval.

### Flat constant-current pulses

The characteristics of the constant current pulses were verified by delivering stimulus pulses into 0.9% NaCl solution, through a twisted-pair bipolar electrode. Flat constant-current pulses could be produced from 20 μA to 2 mA (Figure 3A), with a rise time of 2.8 μS (0–90%; tested at 1 mA). Although active charge-balancing is provided with biphasic stimulation, both monophasic and biphasic pulses are also passively charge-balanced, by grounding the stimulating electrode immediately following a pulse phase (Figure 3B). This feature is programmable, and when used during monophasic stimulation, it leads to a brief reversal of the current direction following a pulse phase, for achieving zero net charge at the electrode-electrolyte interface.

### Stimulation inside the water maze

**Figure 4:**
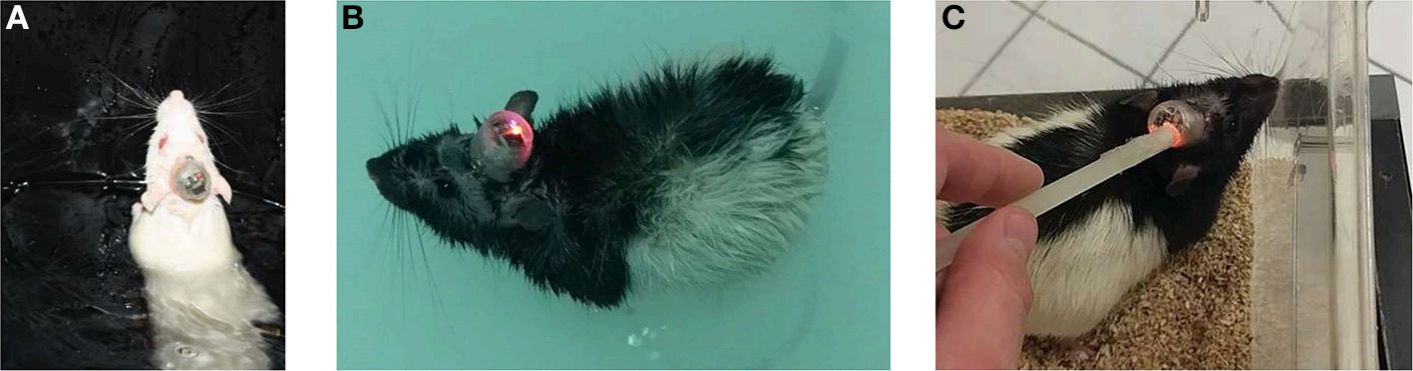
Photographs show the device operating inside the water-maze for Sprague Dawley **(A)** and Long Evans **(B)** rats. Devices were activated inside either the home-cages or the water-maze room, using a magnet **(C)**.

**Figure 5:**
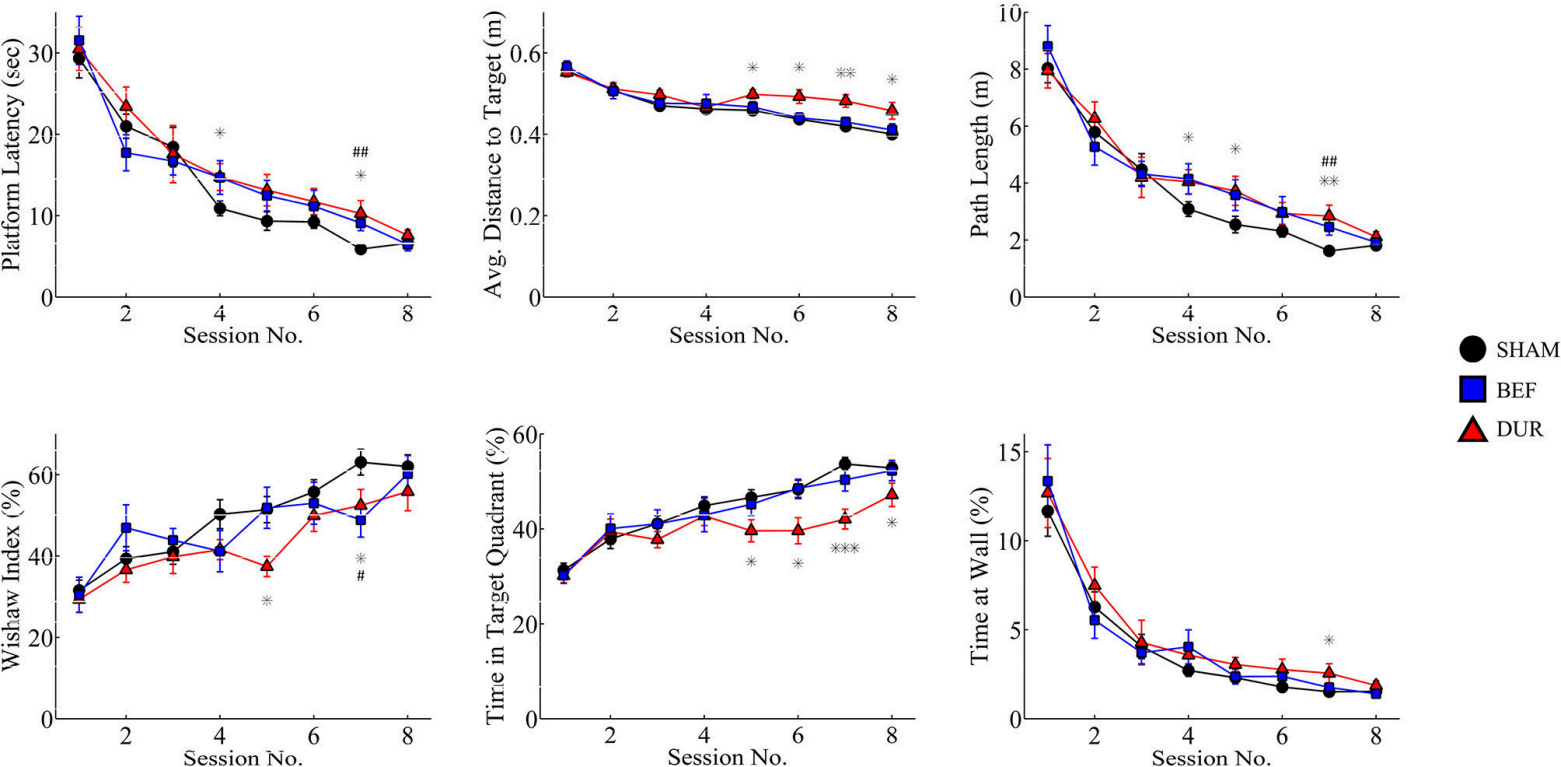
Acquisition data is shown for both stimulated and sham-stimulated rats. Statistical significance is shown for differences between groups for a given session; ^*^p < 0.05; ^**^p < 0.01; ^***^p < 0.001 when comparing DUR vs. SHAM rats, #*p* < 0.05; *##p* < 0.01; *###p* < 0.001 when comparing BEF vs. SHAM rats. Performance deficits are observed mainly in the rats receiving stimulation during the task (DUR), including an increased average distance to target and a reduced time spent in the target quadrant, compared to SHAM controls. This highlights a transient effect of DBS on task performance that is less pronounced in the BEF stimulation group.

The devices had shown to function correctly in every rat and in every trial (>1,400 acquisition trials; 88 probe sessions), and remained operational when rats swam underwater (see Figure 4 for photographs of the device inside the water maze). During acquisition, all rats had demonstrated a robust pattern of learning (Figure 5), as shown by significant effect of test session on both the latency to platform [*F*_(7, 328)_ = 51.89; *p* < 0.0001] and path length [*F*_(7, 328)_ = 64.62; *p* < 0.0001]. Significant group-effects were observed for platform latency [*F*_(2, 248)_ = 3.42; *p* = 0.034], path length [*F*_(2, 328)_ = 4.26; *p* < 0.015], and average distance to target [*F*_(2, 328)_ = 11.96; *p* < 0.0001], Whishaw's Index [a measure of swim efficiency; *F*_(2, 328)_ = 6.49; *p* < 0.0017] and the percentage time in the target quadrant [*F*_(2, 328)_ = 11.7; *p* < 0.0001]. Many of these changes are indicative of performance deficits in the DUR group, as opposed to the BEF group which had shown a performance closer to that of the SHAM group. No significant group difference was observed for thigmotaxis [*F*_(2, 328)_ = 1.99; *p* = 0.14].

**Figure 6:**
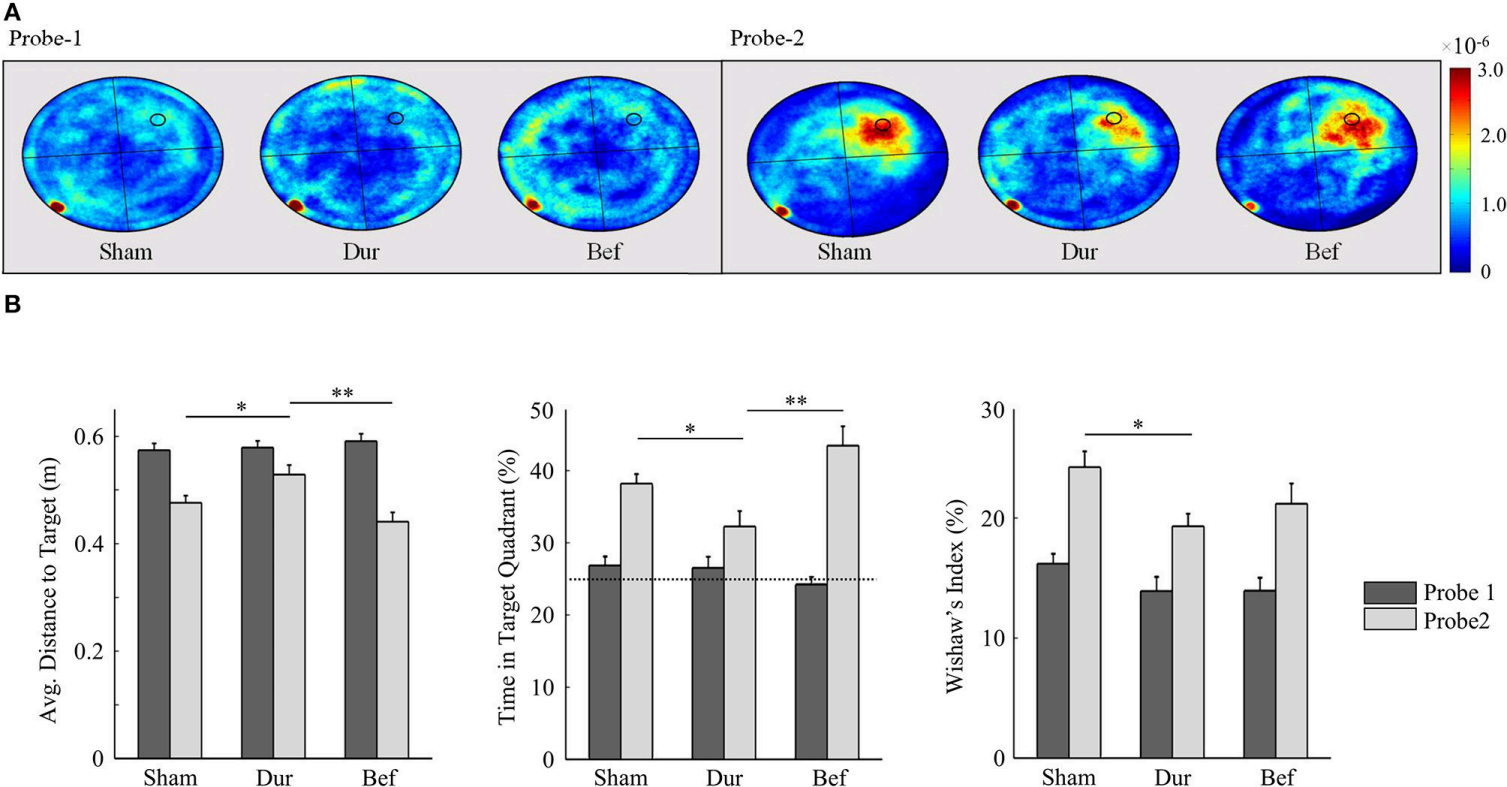
Rat swim paths during the probe sessions are represented as group-normalized 2D-histograms **(A)**. The platform position (during acquisition) is indicated as a small circle in the goal quadrant (NW). All rats had demonstrated an ability to learn the task, as shown by an increased activity in the NW quadrant during the second probe session, as compared to chance-level (25%). The mean distance to the target (**B**-left), the % time in the target quadrant (**B**-center), and the Whishaw's Index (**B**-right) are shown for both probe sessions. ^*^P < 0.05; ^**^p < 0.01.

By the second probe session, all groups had demonstrated a robust memory performance, as highlighted by an increased time in the target quadrant, relative to chance level (Figure 6). Significant group effects were only observed during the second probe session, including the average distance to target [*F*_(2, 41)_ = 6.26; *p* = 0.0042], Whishaw's Index [*F*_(2, 41)_ = 3.48; *p* = 0.04], and the time spent in the target quadrant [*F*_(2, 41)_ = 6.99; *p* = 0.0024]. *Post-hoc t*-tests had shown that DUR rats had demonstrated slight reductions in Whishaw's Index [*t*_(31)_ = 2.59; *p* = 0.014], time in the target quadrant [*t*_(31)_ = 2.55; *p* = 0.016], and a slight increase in the average distance to the target [*t*_(31)_ = 2.37; *p* = 0.024], as compared to SHAM controls. While no significant differences were observed between the BEF and SHAM groups, the BEF group had performed better during than the DUR group during the second probe trial, with regards to the average distance to target [*t*_(21)_ = 3.53; *p* = 0.002] and the time spent in the target quadrant [*t*_(21)_ = 3.26; *p* = 0.0038].

### Group-housing performance

**Figure 7:**
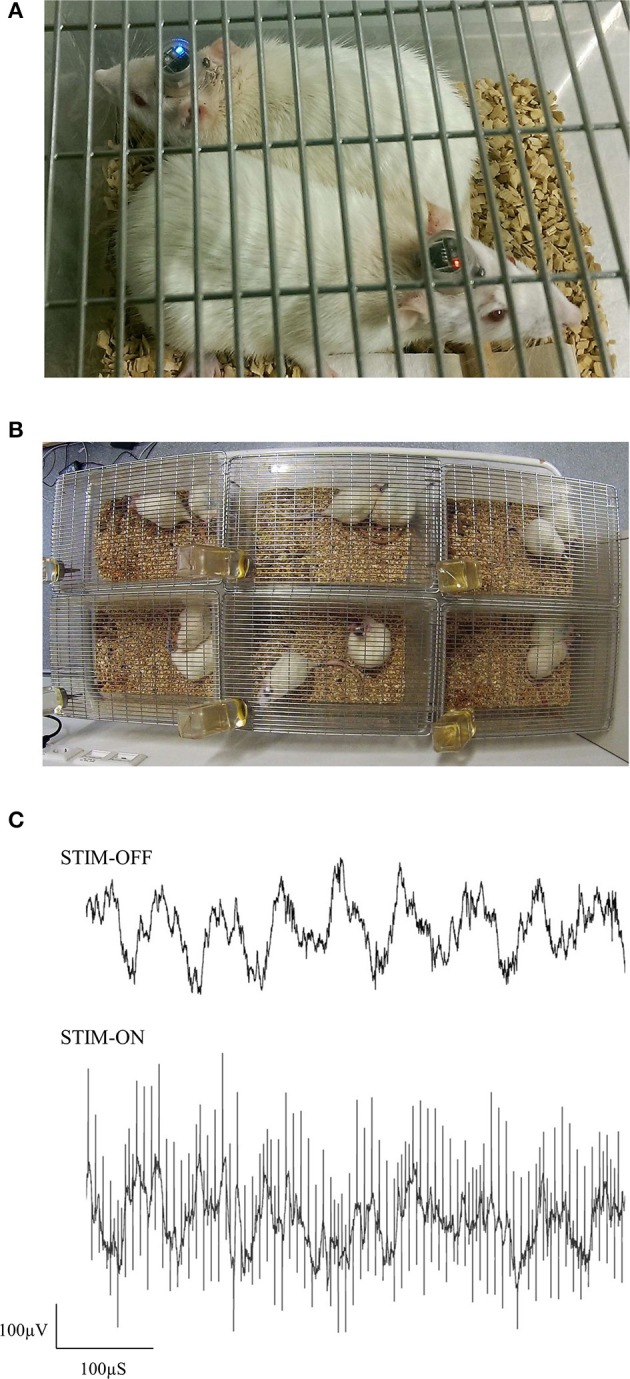
Photographs of pair-housed rats during a stimulation session **(A,B)**. EEG from dCA1 is shown with and without high-frequency stimulus pulses **(C)**. Having an electrically isolated stimulator ensured that only volume conduction artifacts were present in the EEG, due to stimulus pulses propagating through the brain.

In this preliminary study, female Sprague Dawley rats were implanted with electrodes in the ReRh, and were pair-housed for 8 days, following the recovery period (Figures 7A,B). For 5 of these days, rats had received either high-frequency stimulation (130 Hz; 90 μS/phase monophasic or biphasic; 200 μA), or sham-stimulation for 1 h daily, with recordings of prefronto-hippocampal EEG and mobility taken before and after this period. During this period, no obvious malfunctions were observed resulting from the environment or social activities. The use of charge-balanced biphasic pulses is demonstrated during simultaneous EEG recording (Figure 7C), using a commercial wireless system (W32; Multichannel Systems).

## Discussion

A portable stimulator was developed by combining an ultra-small PCB assembly with 3D printing techniques, in order to expand on the range of currently available stimulus paradigms. For its small size (0.8/2.8 g with battery and head-cap), the device offers a high compliance voltage, and the ability to generate charge-balanced biphasic pulses from 2 separate channels (see Table 1 for a comparison with existing devices). This system can also be cheaply produced (<€30 per device), which can allow many rats to be stimulated simultaneously inside their home cages, without having to consider tethered solutions, or alternative housing. This is the first portable stimulator to be utilized in both chronic group-housing and water-maze environments.

### 3D-printed device housing

The 3D-printed head-socket has previously been utilized for the pair-housing of rats, following stereotaxic surgery (Pinnell et al., 2016). While this previous study had utilized a metal thimble as the protective cap, a smaller 3D-printed thimble was designed for the current study that housed the device and its battery. Transparent ABS was chosen for this as it offered the strength to withstand the environment for prolonged periods of time, as well as allowing the device's LED's to be visible during experiments. The 3D-printed device housing had functioned adequately during the pair-housing experiments, and had not sustained any damage resulting from normal rat activities such as grooming or playing. During supervision, rats were not observed to bite or chew the implant of their cage-mates, and no signs of such damage was observed. In addition to the practical and ethical benefits of keeping animals pair-housed inside their home cages, this device can help to enrich DBS studies by potentially ameliorating numerous physiological and behavioral deficits that otherwise pertain to social isolation. Furthermore, this method can pave the way toward novel stimulation paradigms, such as studies that assess the social effects of DBS.

During the water-maze experiments, no signs of leakages or malfunctions were observed in any of the devices, throughout the test period (>1,500 trials). When combined with petroleum jelly, the circular design of the cap and socket was found to be optimal for keeping water away from the cap interior during vigorous pre-experiment waterproof testing. Notably, the devices remained operational when rats swam underwater, which was common during the early stages of training. By allowing stimulation to take place inside a water maze, experimenters have the opportunity to directly observe the acute effects of stimulation on the behavior they are trying to assess. Given the widespread popularity of the MWM as a tool for assessing various aspects of learning and memory, this device can pave the way toward integration of DBS with more complex behaviors. In addition to these benefits, the portable stimulators were found to simplify the execution of the experiment, as compared to previous in-house experiments utilizing a cable tether. Animals could be transferred between the holding and test rooms without changing connectors or manipulating the implant, and DBS could be seamlessly activated at any part of the experiment without touching the rat. Such measures allowed stimulation to be activated/deactivated immediately prior to placing the rats inside the water maze, without any delay periods.

### Stimulator design

From an early design stage, strict size restrictions were placed on the overall size of the device cap/housing, to ensure that it can fit onto a pre-existing head-socket. As such, this had necessitated a 12.5 mm diameter PCB using ultra-small electronic components and high-density circuit design. Some design concessions were made through this process, including the use of variable resistors for setting the constant current intensity, instead of e.g., a digital potentiometer. Since current intensity adjustments were carried out using an oscilloscope, accuracy penalties within the range of ± 3–5 μA were expected. By comparison, existing systems may offer comparatively higher accuracy through e.g., a digital potentiometer (Ewing et al., 2013), or a lower accuracy, due to a constant-current that is dependent on the system's orientation (Millard and Shepherd, 2007) or dependent partly on the electrode and brain impedances (de Haas et al., 2012; Forni et al., 2012).

Many of the design choices with this system reflect functionality over battery life, making this system more suited for acute experiments, or for stimulation sessions lasting up to 30 h. The constant-current generator for instance, had utilized LED's for maintaining a fixed reference voltage, as opposed to standard diodes. This had allowed for a visible feedback of DBS that would vary based on the stimulus parameters. For example, increasing the duty cycle results in an increased LED brightness, and using low-frequency or bursting stimulation causes the LED's to flash. An additional bright LED was included, which could be programmed to flash at any part of the experiment, such as when the system had finished a 30-min stimulation period. Furthermore, this additional LED could be utilized in video-tracking software that supports head-mounted LEDs. Further battery life reductions are a result of the switched-capacitor charge pumps that are used to generate the high compliance voltages. Such voltage amplification is normally omitted from ultra-small devices weighing <5 g (Millard and Shepherd, 2007; Arfin et al., 2009; de Haas et al., 2012; Kouzani et al., 2013, 2016), which instead source the compliance directly from the system's battery. While this method can extend the battery life and reduce the device size, it places a limitation on the maximum stimulation current, and carries the risk of the compliance voltage becoming too low toward the end of the battery's lifetime. An exception to this is where silver oxide batteries are used, which can maintain a relatively stable voltage throughout its lifetime (de Haas et al., 2012). Inadequately designed electrodes, or those that are mishandled during surgery, may become of higher impedance than normal, with the effect of increasing the compliance voltage requirements further. As observed in the present experiments and bench tests, the required voltage will largely depend on the impedance of the electrode that is used; and this can typically vary by up to a few volts, from electrode to electrode. It is of note that although the present device is capable of up to 18 V compliance, this was fixed at the lowest limit of 12 V using a voltage regulator, for ensuring that the compliance voltage level is guaranteed throughout the lifetime of the battery. The regulated 12 V compliance can thus drive up to 2 mA in saline, using the same twisted-pair bipolar stimulating electrodes as those used during the behavioral test session.

Chronic experiments are possible with a single battery, provided that rats undergo fixed daily stimulation sessions, as demonstrated in the current study. Otherwise, the system's battery can be quickly replaced as required. For chronic continuous stimulation, there is the possibility of using a larger battery with a higher capacity. For example, a 3 V CR1/3N battery can theoretically provide up to 5 days of continuous DBS (200 μA, 130 Hz), based on its rated capacity (170 mAh) and increased efficiency; and it can be adapted for use with a slightly larger head-cap enclosure. Such a device would weigh an estimated 4.6 g (including additional ABS for the head cap), and could thus easily be carried on the head of the animal.

### Behavioral effects of thalamic stimulation

The reuniens and rhomboid thalamic nuclei (ReRh) were chosen as part of an ongoing investigation into their role in learning and memory (Cassel et al., 2013; Griffin, 2015). In the present experiment, TBS of the ReRh had shown primarily acquisition deficits, which were mainly observed during the second-half of the acquisition period for rats stimulated during the maze task; yet all rats were nonetheless capable of learning the platform location and displaying a robust memory performance during the probe trial. Notably, stimulated rats were more likely to take an indirect path to the target, which may explain the slight increases in path length, platform latency, and the average distance to the target. Previously, inactivation (Cholvin et al., 2013) or lesion (Dolleman-van der Weel et al., 2009) of this structure was not found to impair acquisition performance on the MWM task, when compared to sham controls. However, strategy changes were highlighted, as either a modification of the search strategy during probe trials (Dolleman-van der Weel et al., 2009), or as an impaired ability for rats to switch from a procedural to a place strategy inside the double-H maze (Cholvin et al., 2013). Theta-burst stimulation parameters have previously been used as an alternative to high or low-frequency parameters, as it has been proposed to better mimic the functional activity of limbic networks. Previously, fornix-TBS has previously been shown to improve memory in rats with either medial-septal muscimol inactivation (Shirvalkar et al., 2010), or traumatic brain injury (Sweet et al., 2014). In the present experiment, TBS of the ReRh could be interfering with the natural theta rhythm in a disruptive way, and affecting the functional cooperation of both the ReRh and the hippocampus; the latter of which is known to be sensitive to the performance of reference memory tasks (Morris, 1983). This could have acute implications, as rats that were stimulated before the maze task (but not during) had displayed an acquisition and probe performance that was more in line with the sham-group. Furthermore, it is known that not only does CA1 receive strong afferent fibers from the ReRh (Wouterlood et al., 1990), but strong excitatory responses are also observed in this region, following ReRh stimulation (Dolleman-Van der Weel et al., 1997). Future experiments that include EEG recordings alongside stimulation may help to build a clearer picture of the functional implications of ReRh stimulation during the behavioral task.

## Data availability

The design files and detailed assembly instructions for the portable devices can be found in the Figshare repository at https://figshare.com/s/31122e0263c47fa5dabd. The datasets generated can also be found at https://figshare.com/s/7ee8888fb8f5cb75ec14. Technical data: LINK, https://figshare.com/s/31122e0263c47fa5dabd; doi: 10.6084/m9.figshare.5975356. Experimental data: LINK, https://figshare.com/s/7ee8888fb8f5cb75ec14; doi: 10.6084/m9.figshare.5280679.

## Author contributions

RP designed, developed, and tested the devices, electrodes and implants. RP designed, executed and analyzed both behavioral experiments. RP wrote the manuscript. JC and AP provided valuable input into the manuscript and the MWM experiment design, and AP assisted with animal upkeep and euthanasia. JC and UH are the project leaders.

### Conflict of interest statement

The authors declare that the research was conducted in the absence of any commercial or financial relationships that could be construed as a potential conflict of interest.
